# Adolescent Gambling, Gambling Expenditure and Gambling–Related Harms in Finland, 2011–2017

**DOI:** 10.1007/s10899-019-09892-7

**Published:** 2019-09-13

**Authors:** Susanna Raisamo, Jaana M. Kinnunen, Lasse Pere, Pirjo Lindfors, Arja Rimpelä

**Affiliations:** 1 Department of Public Health Solutions, Alcohol, Drugs and Addictions Unit, National Institute for Health and Welfare, P.O. Box 30, 00271 Helsinki, Finland; 2 Faculty of Social Sciences, Health Sciences, University of Tampere, Tampere, Finland; 3 PERLA – Tampere Centre for Childhood, Youth and Family Research, University of Tampere, Tampere, Finland; 4 Department of Adolescent Psychiatry, Pitkäniemi Hospital, Nokia, Tampere University Hospital, Tampere, Finland

**Keywords:** Adolescent, Gambling, Harm, Trend, Gambling expenditure

## Abstract

Existing literature on recent trends in adolescent gambling is scarce. The rapidly changing landscape of gambling, together with the generally applied legal age limits, calls for the continuous monitoring of gambling also among the adolescent population. In Finland, the legal gambling age is 18. We examined changes in adolescents’ gambling, gambling expenditure and gambling–related harms from 2011 to 2017. Comparable cross-sectional biennial survey data were collected in 2011, 2013, 2015 and 2017 among 12–18-year-olds (*N* = 18,857). The main measures were self-reported six-month gambling, average weekly gambling expenditure (€) and harms due to gambling. Data were analyzed using cross-tabulations, χ^2^-tests and linear regression analysis. A significant decline in gambling among minors (aged 12–16-year-olds) was found (β = − 0.253), while no significant changes were observed among 18-year-olds (who are not targeted by the law). The mean gambling expenditure also declined from 2011 to 2017. Adolescent gamblers experienced significantly less (*p *= .003) gambling–related harms in 2017 (7.4%) compared to 2011 (13.5%). Adolescent gambling and its related negative consequences have become less prevalent in Finland between 2011 and 2017. Further monitoring is necessary to ascertain whether the positive direction will continue. Also, empirical analyses providing evidence of reasons for the observed trend are warranted.

## Introduction

Adolescence is a unique developmental phase of life, usually characterized as a time of increased involvement in risk behaviours, such as drinking alcohol, substance use and delinquency. Gambling is also considered as a form of risk behaviour in adolescence (Derevensky and Gupta 2004; Messerlian et al. 2007; Tozzi et al. 2013). Different forms of risky behaviour share some common features as they are typically correlated. Involvement in these risky behaviours makes adolescents more vulnerable to adverse consequences and also puts them at risk of developing addictions.

Excessive gambling is considered to be non-substance addictive behaviour (Grant et al. 2010); it has received increasing attention as an emergent social and public health issue in many jurisdictions (Adams et al. 2009; Blinn-Pike et al. 2010; Messerlian et al. 2004, 2005). Most Western countries have set a legal age limit of 18 for gambling. Despite age restrictions, being involved in legalized gambling has been shown to be common among adolescents (Blinn-Pike et al. 2010; Volberg et al. 2010).

Prior studies conducted among adolescents suggest that excessive patterns of gambling may lead to a variety of individual and social harms, such as parent–adolescent relationship conflicts, school-related harms (such as absenteeism), financial harm and even criminal activity (Calado et al. 2017; Raisamo et al. 2013; Splevins et al. 2010). A review conducted by Delfabbro et al. (2016) indicated that between 4 and 8 per cent of adolescent gamblers experienced significant gambling–related problems. Considering that adolescents are more vulnerable than adults to the negative consequences of gambling and that adolescents are two to four times more likely to be problem gamblers than adults (Hardoon and Derevensky 2002), there is a strong case to be made for protecting underage adolescents from gambling and its related harms. It is justifiable for the individual’s health and wellbeing, and it is also justifiable from society’s point of view.

Internationally, adolescent gambling is investigated in numerous papers (for reviews see Blinn-Pike et al. 2010; Calado et al. 2017) and research has grown considerably over the past two decades (Derevensky and Gilbeau 2015). Research has tended to be quantitative, focusing on the prevalence, correlates and risk factors of gambling (Blinn-Pike et al. 2010; Floros 2018). Rarely, attention has been devoted to the time trends of gambling among the adolescent population (Jacobs 2000; Stinchfield 2011). Previous studies on gambling trends have focused on the adult population (e.g. Abbott et al. 2014, 2016; Ludwig et al. 2012; Olason et al. 2015; Welte et al. 2015). Surveillance of gambling trends among the adolescent population, however, is particularly relevant when evaluating gambling policies (e.g. the effects of minimum-age laws) and planning appropriate gambling–related harm-minimization strategies.

Results from the Minnesota Student Survey revealed a gradual and consistent decline in gambling among students in grades 8, 9 and 11 from 1992 to 2016 (Minnesota Department of Human Services 2017; Stinchfield 2011). Volberg et al. (2010) also found similar results. The British Gambling Commission’s survey data (Gambling Commission 2016), collected in England and Wales among 11–15-year-olds, reported that although the prevalence of past-week gambling participation dropped from 22% in 2007 to 16% in 2016, the reported rates of past-week gambling participation for the previous 3 years (2014, 2015, 2016) remained stable. All in all, there are limited data available for addressing the current trends in gambling among adolescents.

The landscape of gambling is changing rapidly in both land-based and online environments and gambling marketing, for example, is ubiquitous (Newall et al. 2019). Arguably, the growing availability and commercialization of gambling are likely to create an environment in which adolescents are highly exposed to gambling (King et al. 2010; Monaghan et al. 2008). Therefore, the changing landscape of gambling calls for continuous monitoring of gambling patterns, also among adolescent population.

In Finland, the setting for the present study, gambling is organized as a governmental monopoly and operated by one legal operator, Veikkaus Oy. Gambling profits are returned to society and used, for example, for funding culture, science, sports, youth and social work, and horse breeding. Past-year population gambling prevalence is high, with approximately 80 per cent of the population gambling and nearly half of them gambling on a weekly basis (Salonen and Raisamo 2015). Finland has ranked fourth in per capita spending on gambling in the world (The Economist 2017) and gambling is very concentrated, as a small group of gamblers (the top 5%) brings in approximately half of the gaming profits (Salonen et al. 2017). The research literature suggests that generally, higher gambling expenditure increases the risk of experiencing harm from gambling (Hansen and Rossow 2008; Markham et al. 2016).

In Finland, the minimum legal gambling age is 18 years old. The age limit for gambling was raised from 15 to 18 years old between 2010 and 2011. From the international perspective, Finland is of particular interest due to an exceptional decentralized system of slot machines, which guarantees easy access to these type of games in supermarkets, kiosks, gas stations, cafés etc. The most preferred type of gambling among Finnish underage adolescents is slot machines, and we have shown in our previous study that, regardless of age restriction, approximately 13% of 12–16-year-olds manage to gamble on slot machines (Raisamo et al. 2015). So far, underage gambling expenditure has rarely been a subject of specific investigation, and therefore, the present study adds to the scarce body of literature in this field as well.

The aim of this paper is to contribute towards the clear lack of empirical data on recent trends in adolescent gambling. More precisely, we examined trends in adolescent gambling, gambling expenditure and gambling–related harm in Finland covering the period from 2011 to 2017.

## Methods

### Participants and Procedure

The data were derived from a nationwide monitoring system of adolescent health and health behaviour in Finland, the Adolescent Health and Lifestyle Surveys (AHLS). The AHLS has been undertaken on a biennial basis among nationally representative samples of 12-, 14-, 16- and 18-year-old Finns since 1977. Questions about gambling have been included since 2011.

Samples were obtained from the Finnish Population Register Centre by selecting all those born on certain days in June, July or August. The mean ages of the respondents in all age groups were approximately the same every survey year in 2011, 2013, 2015 and 2017, namely 12.6, 14.6, 16.6 and 18.6 years. The total number of respondents and response rates are presented in Table 1. Regardless of age, the response rates were generally higher for girls than for boys. In this paper, *the underage population* refers to adolescents aged 12–16 years old; 18-year-olds were analyzed as a comparison group as they were of a legal age to gamble. The overall study procedure, sampling strategies, measures and time of data gathering have been maintained the same over the years, enabling comparisons between the survey years. The data collection has used self-completed questionnaires which have been mailed to adolescents in the samples (with an option to answer online). Two reminders were sent to non-respondents.

**Table 1:** The number of respondents and the response rate (%) by age, gender and survey year in 2011, 2013, 2015 and 2017 according to the Adolescent Health and Lifestyle Survey

Gender and age	Survey year			
	2011	2013	2015	2017
*Boys*				
12 years old	320 (47)	264 (40)	653 (44)	299 (44)
14 years old	621 (45)	417 (31)	905 (41)	572 (41)
16 years old	566 (36)	440 (31)	711 (31)	459 (33)
18 years old	392 (27)	284 (21)	601 (25)	370 (28)
Total	1899 (38)	1405 (30)	2870 (34)	1700 (36)
*Girls*				
12 years old	326 (55)	294 (48)	689 (51)	333 (51)
14 years old	777 (66)	603 (48)	1097 (51)	723 (54)
16 years old	868 (58)	601 (47)	1085 (48)	697 (53)
18 years old	696 (52)	632 (42)	957 (41)	605 (45)
Total	2667 (56)	2130 (46)	3828 (47)	2358 (51)

Total *N* = 18,857

### Measures

*Six*-*month gambling* is based on the question: “During the past 6 months, have you gambled for money?” with the response alternatives: “*No*”, “*Yes, daily or nearly daily*”, “A *couple of times a week*”, “*A couple of times a month*” and “*Rarely*”.

*Gambling expenditure* was measured with the following question: “How much money do you spend on gambling in a week (in €)?” with the response options: “None” and then a space in which to write down an answer in euros. Responses to this item were collapsed into categories of *low expenditure* (0.01–4.99 euros), *medium expenditure* (5–19.99 euros) and *high expenditure* (20 euros or more). Question on gambling expenditure was included in the 2011, 2013 and 2017 surveys.

#### Gambling–Related Harms

In this paper, the term *gambling harm* is used to refer to an eight-item list that asks whether the respondent has experienced any of the following harms: “conflicts with parents”, “conflicts with friends”, “disruptions of daily rhythm”, “disruptions in school/work”, “feeling guilty or ashamed”, “skipping school/work”, “unable to pay debts” and “stealing money for gambling”. Respondents were allowed to tick more than one option. For each harm on the list, respondents were also asked to report the frequency of experiencing harms: (i) *seldom or not at all*, (ii) *about once a month*, (iii) *about once a week*, and (iv) *daily/almost daily*. Following the similar categorization used in an earlier Finnish study (Raisamo et al. 2013), three options (ii, iii, iv) were collapsed into one category of *(the respondent) had experienced harm*. A combined variable based on the eight items was created with three categories: *no harms, one harm* and *more than one harm.* Question on gambling–related harm was included in the 2011 and 2017 surveys.

*Survey year*, *age group* and *gender* were also used in the analyses. Information on age and gender were received from the Finnish Population Register Centre.

### Statistical Analysis

First, six-month gambling participation prevalence was calculated according to survey year for the whole study population and also for gender and age groups. Age-standardized six-month gambling prevalence was calculated for 12–16-year-olds using direct adjustment (i.e. giving equal weights to each group). Among gamblers, means and percentages for gambling expenditure and percentages for having experienced harm due to gambling were calculated for the whole study population and then by gender and age group according to survey year. Linear regression analysis for six-month gambling among 12–16-year-old adolescents was conducted with survey year as a continuous variable. The significance of differences between the survey years was assessed by the Pearson’s χ^2^ test. All analyses were performed using IBM SPSS Statistics V.23 (IBM, Armonk, NY, USA).

### Ethics

The surveys were carried out in accordance with the Declaration of Helsinki and was approved by the Ethics Committee of the Tampere Region. Participation was voluntary. All subjects were informed of the aims and confidentiality of the survey. Filling in and returning the questionnaire was regarded as providing consent.

## Results

### Changes in Six-Month Gambling Prevalence, 2011–2017

**Fig. 1:**
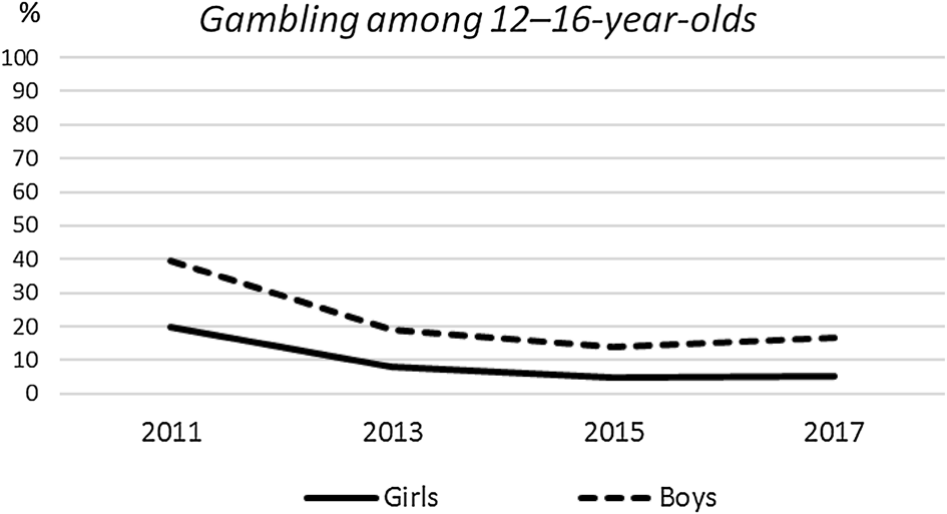
Age-standardized 6-month gambling prevalence (%) among 12–16-year-olds in 2011–2017 according to the Adolescent Health and Lifestyle Survey

Tables 2, 3 and Fig. 1 illustrate the changes in the six-month gambling prevalence from 2011 to 2017 among Finnish adolescents. Over the study period, the prevalence of six-month gambling declined significantly among 12–16-year-old boys and girls but no significant change occurred among 18-year-olds (Table 2, Table 3). Age-standardized six-month gambling prevalence among underage boys dropped from 39% in 2011 to 17% in 2017 and among underage girls from 20 to 5% for the respective years (Fig. 1). The prevalence of six-month gambling remained substantially higher among boys than among girls over the entire study period.

**Table 2:** The six-month gambling participation (%) among adolescents by gender and age group in 2011, 2013, 2015 and 2017 according to the Adolescent Health and Lifestyle Survey

6-month gambling	12–16 years old				18 years old			
	2011	2013	2015	2017	2011	2013	2015	2017
*Boys*								
No gambling	42.9	73.8	81.7	76.4	24.2	27.5	34.6	31.0
Daily/nearly daily	3.7	0.7	0.6	0.8	6.4	5.4	5.2	0.9
Once/twice a week	18.1	3.2	2.0	2.6	26.3	21.1	18.8	21.9
A few times a month	16.6	4.9	4.7	6.2	22.4	27.1	21.6	24.4
Rarely	18.8	17.4	11.0	14.1	20.6	18.9	19.6	17.5
Total % (*n*)	100 (1493)	100 (1109)	100 (2235)	100 (1316)	100 (388)	100 (280)	100 (592)	100 (365)
*p* value	< .001				.07			
*Girls*								
No gambling	69.5	88.7	93.4	92.2	62.0	64.2	66.2	61.3
Daily/nearly daily	0.6	–	0.1	0.1	0.9	–	0.6	0.3
Once/twice a week	2.1	0.3	0.2	0.1	4.3	3.8	3.1	3.5
A few times a month	7.6	1.3	0.8	0.8	9.4	9.6	10.1	11.2
Rarely	20.3	9.6	5.5	6.8	23.5	22.5	19.9	23.7
Total % (*n*)	100 (1950)	100 (1490)	100 (2845)	100 (1738)	100 (695)	100 (628)	100 (953)	100 (599)
*p* value	< .001				.316			
*All*								
No gambling	58.0	82.3	88.2	85.4	48.5	52.9	54.1	49.8
Daily/nearly daily	1.9	0.3	0.3	0.4	2.9	1.7	2.5	2.2
Once/twice a week	9.0	1.6	1.0	1.2	12.2	9.1	9.1	10.5
A few times a month	11.5	2.8	2.5	3.1	14.0	15.0	14.5	16.2
Rarely	19.6	12.9	7.9	9.9	22.4	21.4	19.8	21.4
Total % (*n*)	100 (3443)	100 (2599)	100 (5080)	100 (3054)	100 (1083)	100 (908)	100 (1545)	100 (964)
*p* value	< .001				.085			

The *p* value for differences between survey years is determined by Pearson’s χ^2^ test

**Table 3:** Linear regression for six-month gambling among 12–16-year-old adolescents by survey year according to the Adolescent Health and Lifestyle Survey

	β	95% CI	*SE*	*t* value
All	− 0.253	− 0.051; − 0.045	0.002	− 31.083
Boys	− 0.278	− 0.064; − 0.054	0.003	− 22.664
Girls	− 0.244	− 0.042; − 0.035	0.002	− 22.578

The reference category is survey year 2011; *SE* = standard error

### Changes in Gambling Expenditure, 2011–2017

In general, boys spent more on gambling than girls. The mean gambling expenditure declined from 2011 to 2017 among underage boys, but among girls it rose from 2013 to 2017. The differences between years were statistically significant for 12–16-year-olds but not for 18-year-olds. Spending 20 euros or more per week on gambling remained rare (Table 4).

**Table 4:** Changes in weekly gambling expenditure (€) among gamblers by gender and age group in 2011, 2013 and 2017 according to the Adolescent Health and Lifestyle Survey

Gambling expenditure in a week	12–16 years old			18 years old		
	2011	2013	2017	2011	2013	2017
*Boys*	(n = 813)	(n = 285)	(n = 296)	(n = 290)	(n = 202)	(n = 247)
Mean (*SD*)	3.65 (9.75)	3.42 (30.22)	2.30 (8.83)	4.74 (7.45)	3.28 (4.60)	5.78 (12.69)
None	30.5	62.2	64.8	27.3	27.4	29.6
€0.01–4.99	43.6	25.8	19.1	34.6	42.8	35.2
€5–19.99	23.0	10.6	14.0	31.8	26.9	26.7
€ > 20	2.8	1.4	2.0	6.2	3.0	8.5
*p* value	< .001			.131		
*Girls*	(n = 579)	(n = 167)	(n = 127)	(n = 257)	(n = 221)	(n = 230)
Mean (*SD*)	0.94 (4.51)	0.23 (0.95)	0.37 (1.55)	1.68 (7.33)	0.89 (2.18)	1.41 (3.99)
None	70.3	88.6	90.6	58.5	67.3	63.3
€0.01–4.99	25.6	10.8	4.7	32.6	26.8	24.9
€5–19.99	3.5	0.6	4.7	7.4	5.5	11.4
€ > 20	0.7	–	–	1.6	0.5	0.4
*p* value	< .001			.07		
*All*	(n = 1392)	(n = 452)	(n = 423)	(n = 547)	(n = 423)	(n = 477)
Mean (*SD*)	2.52 (8.10)	2.24 (24.04)	1.72 (7.48)	3.31 (7.54)	2.03 (3.74)	3.67 (9.78)
None	47.1	71.9	72.6	42.0	48.2	45.8
€0.01–4.99	36.1	20.3	14.8	33.6	34.4	30.3
€5–19.99	14.9	6.9	11.2	20.3	15.7	19.3
€ > 20	1.9	0.9	1.4	4.0	1.7	4.6
*p* value	< .001			.051		

An en-dash (–) means *no observed cases*; the *p* value for differences between survey years is determined by Pearson’s χ^2^ test; the question on expenditure was included in 2011, 2013 and 2017 surveys

### Changes in Gambling–Related Harms, 2011–2017

The percentage of underage gamblers reporting having experienced at least one harm due to gambling decreased significantly from 2011 to 2017 (13.5% in 2011 vs. 7.4% in 2017). The same decrease was found among boys and girls, but the decrease was not statistically significant among girls (Table 5).

**Table 5:** Percentage (%) of gamblers reporting having experienced harm due to gambling arranged by gender and age group in 2011 and 2017

	12–16 years old		18 years old	
	2011	2017	2011	2017
*Boys*	(n = 843)	(n = 296)	(n = 294)	(n = 246)
No harms	83.0	91.2	84.0	84.6
One harm	10.0	4.7	8.2	11.4
More than one harm	7.0	4.1	7.8	4.1
*p* value	.003		.103	
*Girls*	(n = 585)	(n = 123)	(n = 262)	(n = 230)
No harms	91.5	95.9	93.5	93.5
One harm	5.0	3.3	4.2	5.7
More than one harm	3.6	0.8	2.3	0.9
*p* value	.185		.359	
*All*	(n = 1428)	(n = 419)	(n = 556)	(n = 476)
No harms	86.5	92.6	88.5	88.9
One harm	7.9	4.3	6.3	8.6
More than one harm	5.6	3.1	5.2	2.5
*p* value	.003		.038	

The Adolescent Health and Lifestyle Survey

The *p* value for differences between survey years is determined by Pearson’s χ^2^ test

Based on the list of harm items presented in the 2011 and 2017 surveys

For both boys and girls aged 12 to 16 years, the most prevalent type of harm was “feeling guilty or ashamed”. “Disruptions of daily rhythm” and “disruptions in school/work” were the second most common harm items endorsed by underage girls, whereas among underage boys, “conflicts with friends” was the second most common harm. There were few significant changes in reported harm across time. Among 12–16-year-old boys, “conflicts with parents”, “disruptions in school/work” and “feeling guilty or ashamed” were significantly lower in 2017 compared with 2011 (Table 6).

**Table 6:** The type of gambling harm reported among gamblers (%) by gender and age group in 2011 and 2017

Type of harm	12–16 years old			18 years old		
	2011	2017	*p*	2011	2017	*p*
*Boys*						
Conflicts with parents	5.3	1.0	*.001*	3.1	2.4	*.661*
Conflicts with friends	3.8	3.0	*.548*	4.1	1.6	*.094*
Disruptions of daily rhythm	3.1	2.0	*.344*	3.1	2.4	*.661*
Disruptions in school/work	4.0	1.0	*.012*	4.1	2.0	*.174*
Feeling guilty or ashamed	8.4	4.7	*.038*	11.2	11.8	*.838*
Skipping school/work	3.1	1.7	*.204*	2.7	1.2	*.219*
Unable to pay debts	2.3	1.0	*.182*	1.0	0.8	*.802*
Stealing money for gambling	2.4	0.7	*.068*	1.0	0.0	*.112*
*Girls*						
Conflicts with parents	2.6	0.8	*.235*	0.4	0.0	*.348*
Conflicts with friends	2.6	0.0	*.073*	2.3	0.4	*.083*
Disruptions of daily rhythm	1.9	1.6	*.849*	0.8	0.0	*.184*
Disruptions in school/work	2.7	1.6	*.478*	0.8	0.0	*.184*
Feeling guilty or ashamed	4.6	1.6	*.128*	4.2	6.5	*.250*
Skipping school/work	2.1	0.8	*.352*	1.1	0.0	*.104*
Unable to pay debts	0.7	0.0	*.358*	0.0	0.0	–*sra*
Stealing money for gambling	1.5	0.0	*.166*	0.0	0.4	*.285*
*All*						
Conflicts with parents	4.2	1.0	*.001*	1.8	1.3	*.486*
Conflicts with friends	3.3	2.1	*.230*	3.2	1.1	*.018*
Disruptions of daily rhythm	2.6	1.9	*.426*	2.0	1.3	*.366*
Disruptions in school/work	3.5	1.2	*.015*	2.5	1.1	*.080*
Feeling guilty or ashamed	6.9	3.8	*.023*	7.9	9.2	*.446*
Skipping school/work	2.7	1.4	*.147*	2.0	0.6	*.062*
Unable to pay debts	1.6	0.7	*.172*	0.5	0.4	*.783*
Stealing money for gambling	2.0	0.5	*.030*	0.5	0.2	*.396*

Statistically significant values (*p* < 0.05) are given in italic

The Adolescent Health and Lifestyle Survey

The *p* value for differences between survey years is determined by Pearson’s χ^2^ test

Based on the list of harm items presented in the 2011 and 2017 surveys

## Discussion and Conclusions

Our study provided an overview of recent trends in adolescent gambling in Finland. We found a consistent and significant decline in minors’ gambling from 2011 to 2017, which was accompanied by a decrease in both self-reported gambling–related harms and gambling expenditure. Among the respondents aged 18, the prevalence of gambling, gambling–related harms and weekly gambling expenditure remained relatively unchanged.

Monitoring gambling trends is of little value without understanding the potential contributing factors. Empirical analyses providing evidence of reasons for the observed trends in gambling are lacking in Finland. Therefore, we can only speculate about the factors potentially contributing to the decreasing rates of adolescent gambling. The legislation prohibiting under 18-year-olds from gambling is a concrete example of a gambling policy measure dictating that gambling should not be a part of the lives of adolescents. It is most likely that raising the legal age limit of gambling from 15 to 18 years in 2010–2011 contributed to the overall downward trend in gambling and appeared to be beneficial for minimizing gambling–related harms and expenditures. These findings support well the ultimate aim of the Finnish monopoly system, which is to prevent and decrease the social, financial and health-related harms caused by gambling. On the other hand, no changes were observed among the age group of 18 that is not targeted by the law.

It is important to note that, despite the favourable trend, many underage adolescents (particularly boys) reported gambling. Monitoring compliance with the age limit more strictly, particularly regarding slot machine gambling, may ensure that adolescent gambling rates can be even further decreased in the near future. The responsibility for the age limit monitoring lies with the gaming operator, as well as with the sellers of the games. Nonetheless, the enforcement of the law varies a lot within the premises in which slot machines are available (Warpenius et al. 2016) and some of the gambling reported among minors in our study may reflect this.

Since there are only a few published studies available about recent adolescent gambling trends, it is difficult to make comparisons with other studies. However, a decreased tendency to participate in gambling does not seem to be limited to Finland. Similar patterns in adolescents have been reported, for example, in the UK (Gambling Commission 2016) and in the USA (Minnesota Department of Human Services 2017). Thus, the results of the present study may also reflect explanations other than policy explanations.

These results can also be (cautiously) reflected in the similar changes in other risky behaviours among adolescents, like smoking and drinking. In Finland, smoking, heavy episodic drinking and alcohol use in general have decreased during the 21st century (Kinnunen et al. 2017; Raitasalo et al. 2015). The theories of socio-cultural factors point out in particular how perceptions and attitudes of various norm-breaking behaviours among adolescents have become tighter (Bhattacharya 2016). Furthermore, the pressure for self-control and health consciousness in contemporary culture affects adolescents’ behaviour and self-representations (Bhattacharya 2016). In addition, there has also been a declining trend in lawbreaking behaviour, like stealing and doing damage, during the last decade (Kivivuori et al. 2018).

Nowadays, young people are growing up with information and communication technology embedded in their daily lives, and the role of social media and digital games is heightened (Statistics Finland 2017). Social media also plays a critical role in friendships and in making new friends. Online gaming in particular is one of the most common digital venues for meeting friends: a critical element in the lives of adolescents (Lenhart et al. 2015). Therefore, a growing tendency to adopt other types of leisure-time activities than gambling may have taken place.

### Limitations

Our findings are subject to limitations. First, the data analyzed here were based on self-reports in cross-sectional study designs. Response rates were lower in boys than in girls and underreporting or non-response bias cannot be ruled out. Although our study has a relatively short timescale (2011–2017), it is valuable in describing current trends during the situation in which the gambling market has changed substantially. Due to space limitations in the questionnaire, we were compelled to shape a short question for assessing potential harms due to gambling, and thus, we were not able to use any validated instruments to measure the problem-gambling prevalence rate. We understand that the measure we used does not adequately assess all potential domains of harm. Nonetheless, the formulation and selection of harm items were based on a review of empirical literature regarding items generally included in adolescent problem-gambling screening instruments (Stinchfield 2010). In spite of these limitations, highly comparable surveys with similar samples and data collection procedures give strength to these findings.

## References

[CR1] Abbott MW, Romild U, Volberg RA (2014). Gambling and problem gambling in Sweden: Changes between 1998 and 2009. Journal of Gambling Studies.

[CR2] Abbott M, Stone CA, Billi R, Yeung K (2016). Gambling and problem gambling in Victoria, Australia: Changes over 5 years. Journal of Gambling Studies.

[CR3] Adams PJ, Raeburn J, de Silva K (2009). A question of balance: Prioritizing public health responses to harm from gambling. Addiction.

[CR4] Bhattacharya, A. (2016). *Youthful abandon. Why are young people drinking less?* An Institute of Alcohol Studies Report. July 2016. London: The Institute of Alcohol Studies. http://www.ias.org.uk/uploads/pdf/ias%20reports/rp22072016.pdf. Accessed 25 Oct 2018.

[CR5] Blinn-Pike L, Worthy SL, Jonkman JN (2010). Adolescent gambling: A review of an emerging field of research. Journal of Adolescent Health.

[CR6] Calado F, Alexandre J, Griffiths MD (2017). Prevalence of adolescent problem gambling: A systematic review of recent research. Journal of Gambling Studies.

[CR7] Delfabbro P, King DL, Derevensky JL (2016). Adolescent gambling and problem gambling: Prevalence, current issues, and concerns. Current Addiction Reports.

[CR8] Derevensky JL, Gilbeau L (2015). Adolescent gambling: Twenty-five years of research. Canadian Journal of Addiction.

[CR9] Derevensky JL, Gupta R (2004). Gambling problems in youth: Developmental and applied perspectives.

[CR10] Floros GD (2018). Gambling disorder in adolescents: Prevalence, new developments, and treatment challenges. Adolescent Health, Medicine and Therapeutics.

[CR11] Gambling Commission. (2016). *Young people and gambling 2016. A research study among 11*–*15 year olds in England and Wales*. November 2016. Birmingham: Gambling Commission. https://www.gamblingcommission.gov.uk/PDF/survey-data/Young-people-and-gambling-2016.pdf. Accessed 12 Oct 2018.

[CR12] Grant JE, Potenza MN, Weinstein A, Gorelick DA (2010). Introduction to behavioral addictions. American Journal of Drug and Alcohol Abuse.

[CR13] Hansen M, Rossow I (2008). Adolescent gambling and problem gambling: Does the total consumption model apply?. Journal of Gambling Studies.

[CR14] Hardoon KK, Derevensky JL (2002). Child and adolescent gambling behavior: Current knowledge. Clinical Child Psychology and Psychiatry.

[CR15] Jacobs DF (2000). Juvenile gambling in North America: An analysis of long term trends and future prospects. Journal of Gambling Studies.

[CR16] King D, Delfabbro P, Griffiths M (2010). The convergence of gambling and digital media: Implications for gambling in young people. Journal of Gambling Studies.

[CR17] Kinnunen, J. M., Pere, L., Raisamo, S., Katainen, A., Ollila, H., & Rimpelä, A. (2017). *Nuorten terveystapatutkimus 2017. Nuorten tupakkatuotteiden ja päihteiden käyttö sekä rahapelaaminen*. Sosiaali- ja terveysministeriön raportteja ja muistioita 2017:28. [The Adolescent Health and Lifestyle Survey 2017: Adolescent Smoking, Alcohol Use and Gambling. Reports and Memorandums of the Ministry of Social Affairs and Health 2017:28.] Helsinki: Ministry of Social Affairs and Health.

[CR18] Kivivuori, J., Aaltonen, M., Näsi, M., Suonpää, K., & Danielsson, P. (2018). *Kriminologia. Rikollisuus ja kontrolli muuttuvassa yhteiskunnassa*. [Criminology: Criminality and Control in a Changing Society.] Helsinki: Gaudeamus.

[CR19] Lenhart, A., Smith, A., Anderson, M., Duggan, M., & Perrin A. (2015). *Teens, Technology and Friendships. Video games, social media and mobile phones play an integral role in how teens meet and interact with friends*. August 2015. Washington, DC: Pew Research Center. http://www.pewresearch.org/wp-content/uploads/sites/9/2015/08/Teens-and-Friendships-FINAL2.pdf. Accessed 25 Oct 2018.

[CR20] Ludwig M, Kraus L, Müller S, Braun B, Bühringer G (2012). Has gambling changed after major amendments of gambling regulations in Germany? A propensity score analysis. Journal of Behavioral Addictions.

[CR21] Markham F, Young M, Doran B (2016). The relationship between player losses and gambling–related harm: Evidence from nationally representative cross-sectional surveys in four countries. Addiction.

[CR22] Messerlian C, Derevensky J, Gupta R (2004). Public health perspective for youth gambling. International Gambling Studies.

[CR23] Messerlian C, Derevensky J, Gupta R (2005). Youth gambling problems: A public health perspective. Health Promotion International.

[CR24] Messerlian C, Gillespie M, Derevensky JL (2007). Beyond drugs and alcohol: Including gambling in a high-risk behavioural framework. Paediatrics and Child Health.

[CR25] Minnesota Department of Human Services. (2017). *Problem gambling: A report on the state’s progress in addressing the problem of compulsive gambling and on the percentage of gambling revenues that come from problem gamblers*. February 2017. St. Paul, MN: Minnesota Department of Human Services, Alcohol and Drug Abuse Division. https://mn.gov/dhs/assets/2017-01-gambling-report_tcm1053-275361.pdf. Accessed 12 Oct 2018.

[CR26] Monaghan S, Derevensky J, Sklar A (2008). Impact of gambling advertisements and marketing on children and adolescents: Policy recommendations to minimise harm. Journal of Gambling Issues.

[CR27] Newall PWS, Moodie C, Reith G, Stead M, Critchlow N, Morgan A, Dobbie F (2019). Gambling marketing from 2014 to 2018: A literature review. Current Addiction Reports.

[CR28] Olason DT, Hayer T, Brosowski T, Meyer G (2015). Gambling in the mist of economic crisis: Results from three national prevalence studies from Iceland. Journal of Gambling Studies.

[CR29] Raisamo S, Halme J, Murto A, Lintonen T (2013). Gambling–related harms among adolescents: A population-based study. Journal of Gambling Studies.

[CR30] Raisamo S, Warpenius K, Rimpelä A (2015). Changes in minors’ gambling on slot machines in Finland after the raising of the minimum legal gambling age from 15 to 18 years: A repeated cross-sectional study. Nordic Studies on Alcohol and Drugs.

[CR31] Raitasalo, K., Huhtanen, P., & Miekkala, M. (2015). *Nuorten päihteiden käyttö 1995*–*2015. ESPAD* -*tutkimusten tulokset.* Raportti 19/2015. [Alcohol and Drug Use among Adolescents in Finland 1995–2015: ESPAD survey results. Report 19/2015.] Helsinki: National Institute for Health and Welfare (THL).

[CR32] Salonen AH, Kontto J, Alho H, Castrén S (2017). Suomalaisten rahapelikulutus – keneltä rahapeliyhtiöiden tuotot tulevat? [Finnish gambling expenditure – from whom do the gaming profits come?]. Yhteiskuntapolitiikka.

[CR33] Salonen, A., & Raisamo, S. (2015). *Suomalaisten rahapelaaminen 2015. Rahapelaaminen, rahapeliongelmat ja rahapelaamiseen liittyvät asenteet ja mielipiteet 15*–*74*-*vuotiailla*. Raportti 16/2015. [Finnish gambling 2015: Gambling, Gambling Problems, and Attitudes and Opinions on Gambling Among Finns Aged 15–74. Report 16/2015.] Helsinki: National Institute for Health and Welfare (THL).

[CR34] Splevins K, Mireskandari S, Clayton K, Blaszczynski A (2010). Prevalence of adolescent problem gambling, related harms and help-seeking behaviours among an Australian population. Journal of Gambling Studies.

[CR35] Statistics Finland. (2017). *Digipelaaminen 2017*. [Digital gaming in 2017]. Helsinki. http://tilastokeskus.fi/til/vpa/2017/02/vpa_2017_02_2019-01-31_tie_001_fi.html. Accessed 22 Aug 2019.

[CR36] Stinchfield R (2010). A critical review of adolescent problem gambling assessment instruments. International Journal of Adolescent Medicine and Health.

[CR37] Stinchfield R (2011). Gambling among Minnesota public school students from 1992 to 2007: Declines in youth gambling. Psychology of Addictive Behaviors.

[CR38] The Economist. (2017). The world’s biggest gamblers.

[CR39] Tozzi L, Akre C, Fleury-Schubert A, Surís J-C (2013). Gambling among youths in Switzerland and its association with other addictive behaviours. Swiss Medical Weekly.

[CR40] Volberg RA, Gupta R, Griffiths MD, Ólason DT, Delfabbro P (2010). An international perspective on youth gambling prevalence studies. International Journal of Adolescent Medicine and Health.

[CR41] Warpenius K, Holmila M, Raitasalo K (2016). Compliance with the legal age limits for alcohol, tobacco and gambling: A comparative study on test purchasing in retail outlets. Drugs: Education, Prevention and Policy.

[CR42] Welte JW, Barnes GM, Tidwell MC, Hoffman JH, Wieczorek WF (2015). Gambling and problem gambling in the United States: Changes between 1999 and 2013. Journal of Gambling Studies.

